# *Lactobacillus Intestinalis* Primes Epithelial Cells to Suppress Colitis‐Related Th17 Response by Host‐Microbe Retinoic Acid Biosynthesis

**DOI:** 10.1002/advs.202303457

**Published:** 2023-11-20

**Authors:** Qi‐Wen Wang, Ding‐Jia‐Cheng Jia, Jia‐Min He, Yong Sun, Yun Qian, Qi‐Wei Ge, Ya‐Dong Qi, Qing‐Yi Wang, Ying‐Ying Hu, Lan Wang, Yan‐Fei Fang, Hui‐Qin He, Man Luo, Li‐Jun Feng, Jian‐Min Si, Zhang‐Fa Song, Liang‐Jing Wang, Shu‐Jie Chen

**Affiliations:** ^1^ Department of Gastroenterology Sir Run Run Shaw Hospital Zhejiang University Hangzhou Zhejiang 310058 China; ^2^ Department of Gastroenterology Second Affiliated Hospital of Zhejiang University School of Medicine Hangzhou Zhejiang 310058 China; ^3^ Department of Gastroenterology and Hepatology Shenzhen University General Hospital Shenzhen Guangdong 518055 China; ^4^ Department of Nutrition Sir Run Run Shaw Hospital Zhejiang University Hangzhou Zhejiang 310058 China; ^5^ Department of Colorectal Surgery Sir Run Run Shaw Hospital Zhejiang University Hangzhou Zhejiang 310058 China; ^6^ Institution of Gastroenterology Zhejiang University Hangzhou Zhejiang 310058 China; ^7^ Prevention and Treatment Research Center of Senescent Disease Zhejiang University School of Medicine Hangzhou Zhejiang 310058 China

**Keywords:** colitis, microbes, retinoic acid, Th17

## Abstract

Gut microbiome is integral to the pathogenesis of ulcerative colitis. A novel probiotic *Lactobacillus intestinalis* (*L. intestinalis*) exerts a protective effect against dextran sodium sulfate‐induced colitis in mice. Based on flow cytometry, colitis‐associated Th17 cells are the target of *L. intestinalis*, which is supported by the lack of protective effects of *L. intestinalis* in T cell‐null *Rag1* ^−/−^ mice or upon anti‐IL‐17‐A antibody‐treated mice. Although *L. intestinalis* exerts no direct effect on T cell differentiation, it decreases C/EBPA‐driven gut epithelial SAA1 and SAA2 production, which in turn impairs Th17 cell differentiation. Cometabolism of *L. intestinalis* ALDH and host ALDH1A2 contributed to elevated biosynthesis of retinoic acid (RA), which accounts for the anti‐colitis effect in RAR‐α ‐mediated way. In a cohort of ulcerative colitis patients, it is observed that fecal abundance of *L. intestinalis* is negatively associated with the C/EBPA‐SAA1/2‐Th17 axis. Finally, *L. intestinalis* has a synergistic effect with mesalazine in alleviating murine colitis. In conclusion, *L. intestinalis* and associated metabolites, RA, have potential therapeutic effects for suppressing colonic inflammation by modulating the crosstalk between intestinal epithelia and immunity.

## Graphical abstract

By improving retinoic acid synthesis, a novel probiotic *L. intestinalis* exerts a protective effect against colitis. Retinoic acid triggers epithelial gene alteration, including SAA1, SAA2, and C/EBPA, to downregulate RORγt^+^ Th17 cells. *L. intestinalis* and its associated metabolite, retinoic acid, have potential therapeutic effects for suppressing colonic inflammation.

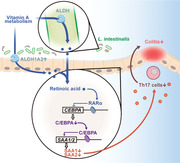

## 1 Introduction

Increasing evidence suggests a causal relationship between commensal microbiota and host health and disease. Gut dysbiosis influences human ulcerative colitis (UC) and experimental murine colitis. It is widely accepted that microbial‐based therapies have the potential to provide individualized treatment for UC patients.^[^ ^1^ ^]^ The modulation of host immunity response by microbiome has been implicated in colitis relief.^[^ ^2^, ^3^ ^]^ However, a deeper understanding of bacterial‐driven gut immunomodulation is required.

T helper type 17 (Th17) cells, a subset of CD4^+^ T cells, exacerbate inflammation‐related disorders. Th17 cells contribute to UC pathogenesis via the secretion of proinflammatory effector cytokines, such as interleukin (IL)−17A, IL‐17F, and IL‐22.^[^ ^4^ ^]^ Emerging studies have shown that bacteria‐derived biomolecules or metabolites could regulate Th17 cells.^[^ ^5^, ^6^ ^]^ In most cases in vivo, however, an intact intestinal epithelial barrier separates some biomolecules and metabolites from immunocytes residing in the LP Thus, intestinal epithelial cells (IECs) are critical in sensing and transducing microbial signals.^[^ ^7^, ^8^ ^]^

Secreted by immunocytes and epithelial cells, serum amyloid A family (SAA) trigger acute‐phase response, and are related to chronic inflammatory diseases and Th17 response.^[^ ^9^, ^10^ ^]^ Administration with antibiotics alters intestinal SAA expression,^[^ ^11^ ^]^ indicating potential involvement of microbiota in intestinal epithelial SAA secretion. Recently, the pathological effects of extrahepatic local SAA have been gaining wider attention, such as in experimental autoimmune encephalomyelitis and cancer.^[^ ^12^, ^13^ ^]^ Thus far, studies focused on the SAA regulation in the liver or small intestine.^[^ ^14^ ^]^ However, the regulation of colonic SAA in colitis remains largely unexplored.

Gut microbes with unique genes could expand the metabolic repertoire of their host by producing numerous metabolites.^[^ ^15^ ^]^ Retinoids, including retinol, retinal and retinoic acid (RA), are key regulators of various physiological processes, which attenuate inflammation and promote mucosal healing in murine colitis models,^[^ ^16^ ^]^ and UC patients.^[^ ^17^ ^]^ RA is a primary mediator of the biological function of vitamin A by directly activating retinoic acid receptors (RARs).^[^ ^18^ ^]^ Microbiota have been reported to contribute to vitamin A metabolism.^[^ ^19^ ^]^ To biosynthesize RA, vitamin A (retinol) is oxidized to retinal by alcohol dehydrogenase, followed by irreversible metabolism to RA by aldehyde dehydrogenases (ALDH).^[^ ^20^ ^]^ ALDH superfamily consists of evolutionarily conserved genes that can be found in most lifeforms, including humans, mice, and some bacteria. However, not all ALDH‐expressing microorganisms possess the capacity to synthesize RA, due to differences in their substrate preference and catalytic activity. Thus, host‐microbial cometabolism could modulate colonic and fecal RA levels.

Here, we identified *Lactobacillus intestinalis* (*L. intestinalis*) as a novel protective bacterium that promotes intestinal homeostasis. *L. intestinalis* supplementation attenuated colitis and downregulated the levels of RORγt^+^ Th17 cells in the colon. Mechanistically, *L. intestinalis* enhanced RA levels through collaborative metabolism with the host colon, which triggered gene expression cascades in epithelial cells to modulate immunity. In the background of the human microbiome, *L. intestinalis* also promoted RA synthesis. And the correlation between *L. intestinalis* and related genes was demonstrated in UC patients. Cumulatively, our results indicate *L. intestinalis* as a promising probiotic against colitis.

## 2 Results

### 2.1 Colonization with *L. Intestinalis* Alleviates Dextran Sodium Sulfate (DSS)‐Induced Colitis

**Figure 1:**
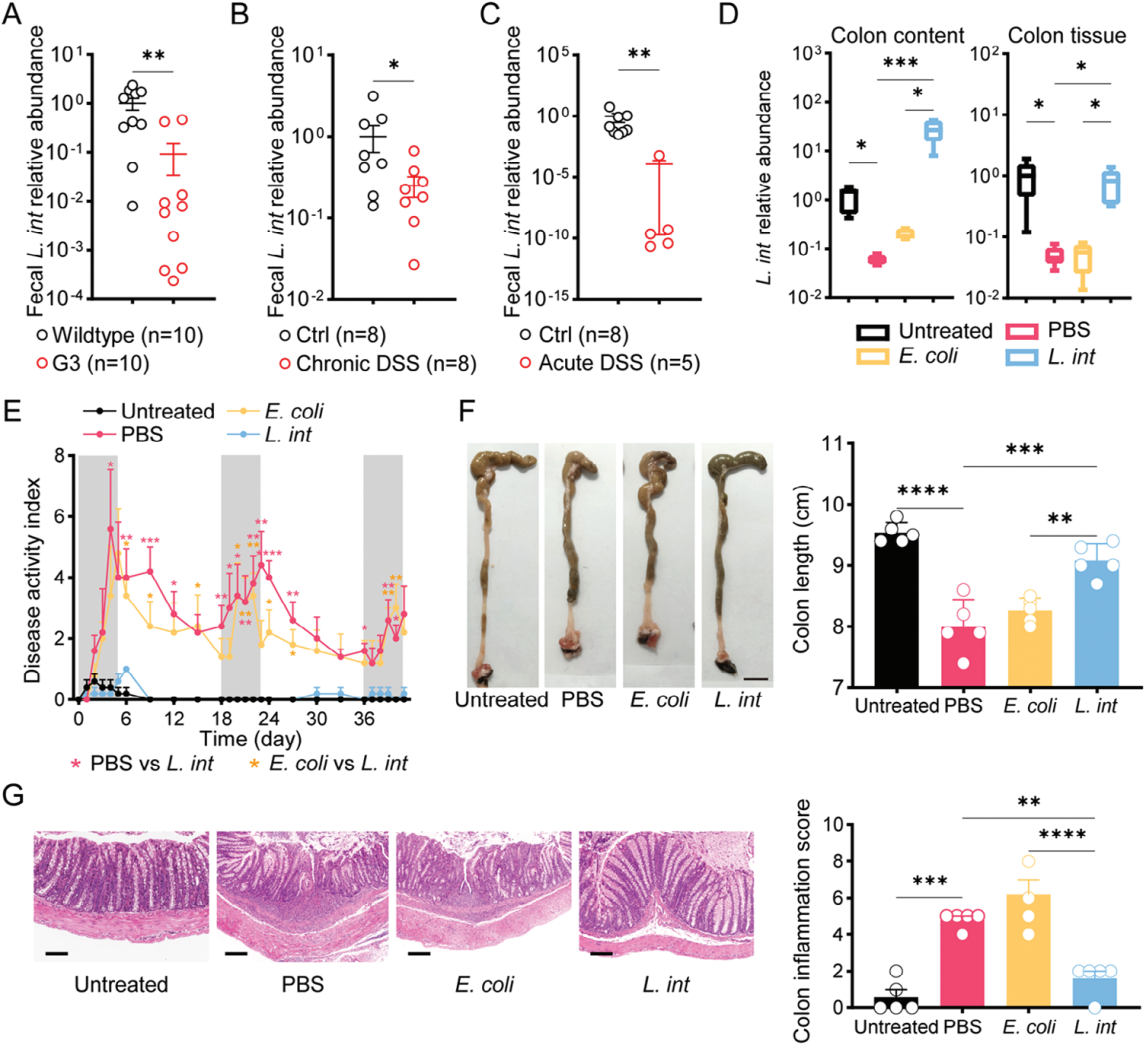
*L. intestinalis* relieved DSS‐induced chronic colitis. A) Fecal abundance of *L. intestinalis* was measured in wild‐type and G3 mice. B,C) Fecal abundance of *L. intestinalis* was measured in a control group, and murine DSS‐induced chronic colitis (B) or acute colitis (C). D) Fecal abundance of *L. intestinalis* was compared among mice treated without (Untreated), or with DSS accompanied by PBS, *E. coli*, or *L. intestinalis* (*L. int*) gavage respectively (*n* = 5). E–G) The pathology of colitis was evaluated among the control group (Untreated) and the three chronic colitis groups with PBS, *E. coli*, or *L. intestinalis* (*L. int*) gavage respectively by disease activity index (E), colon *length* (scale bar, 1 cm) (F) and histological score (scale bar, 100 µm) (G) (*n* = 5). Error bars indicate mean ± SEM. ^*^ *p* < 0.05; ^**^ *p* < 0.01; ^***^ *p* < 0.001; ^****^ *p* < 0.0001. *p* values were based on Mann–Whitney test and one‐way ANOVA with post‐hoc test.

*Terc*‐knockout progeria mouse of the third generation (G3 mice) harbors dysfunctional intestinal homeostasis.^[^ ^21^ ^]^ Our 16S ribosomal RNA (16S rRNA) sequencing analyses revealed that the abundance of *L. intestinalis* was most significantly depleted in G3 mice as compared to the wildtype littermates, which was confirmed by qRT‐PCR (**Figure** **1**A). Consistently, the fecal abundance of *L. intestinalis* decreased in both DSS‐induced acute and chronic colitis (Figure 1B,C).

We then evaluated the pathological changes of DSS‐induced colitis in mice with daily gavage of PBS, *Escherichia coli* MG1655 (*E. coli*), or live *L. intestinalis* (Figure S1A, Supporting Information). Levels of *L. intestinalis* markedly increased in feces and colon tissues, suggesting the successful colonization of gavaged *L. intestinalis* (Figure 1D). As compared to PBS or *E. coli* groups, mice gavaged with *L. intestinalis* had reduced disease activity index (DAI) score (Figure 1E), longer colon length (Figure 1F), and lower spleen weight (Figure S1B, Supporting Information), indicating reduced colitis severity and systemic inflammation. Histological analysis confirmed the significant alleviation of colitis in *L. intestinalis* group (Figure 1G). The expression of inflammation markers, including inducible nitric oxide synthase (*iNOS*) and prostaglandin‐endoperoxide synthase 2 (*Ptgs2*) were downregulated in response to *L. intestinalis* (Figure S1C, Supporting Information). DSS‐induced colitis resulted in the disruption of the gut mucosal barrier, as evidenced by Alcian blue and Periodic acid Schiff (AB‐PAS) stain, as well as the loss of mucin‐secreting goblet cells and downregulation of tight junction proteins (Occludin and ZO‐1); whereas supplement with *L. intestinalis* protected gut mucosal barrier against DSS‐induced damage (Figure S1D, Supporting Information). In line with the above observations, *L. intestinalis* ameliorated DSS‐induced acute colon damage (Figure S2A–E, Supporting Information).

### 2.2 Th17 Immune Response Plays a Role in the Suppressive Effect of *L. Intestinalis*

**Figure 2:**
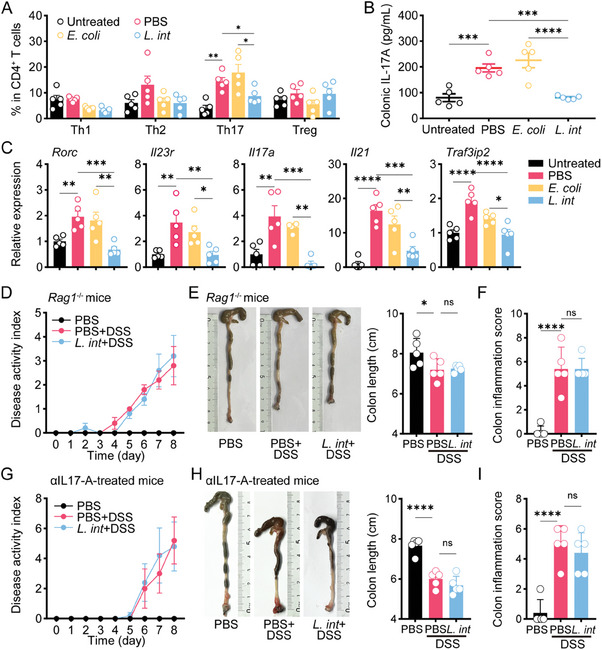
*L. intestinalis* relieved colitis in a Th17‐dependent way. A–C) Frequencies of four CD4^+^ T subsets were analyzed by multicolor flow cytometry in colon LP of untreated mice and chronic DSS‐treated mice with gavage of PBS, *E. coli*, or *L. intestinalis* (*L. int*) (A). Th17 response in colon tissue was analyzed by ELISA (B) and expression of Th17‐related genes (C). D–F) The pathology of colitis was evaluated among *Rag1* ^−/−^ mice, which were treated by acute DSS with gavage of PBS or *L. intestinalis* (*L. int*) or only treated by PBS gavage, by disease activity index (D), colon length (E), and pathological scores (F). G‐I) The pathology of colitis and systemic inflammation was evaluated among αIL17‐A‐treated mice, which were treated by acute DSS with gavage of PBS or *L. intestinalis* (*L. int*) or only treated by PBS gavage, by disease activity index (G), colon length (H), and pathological scores (I). *n* = 5. Error bars indicate mean ± SEM. ^*^ *p* < 0.05; ^**^ *p* < 0.01; ^***^ *p* < 0.001; ^****^ *p* < 0.0001. *p* values were based on one‐way ANOVA with post‐hoc test.

Given close interaction between gut microbiota community and immune response,^[^ ^22^ ^]^ we investigated the immunomodulatory effects of *L. intestinalis* in murine colitis. Flow cytometry analysis of immune cell populations in the colonic LP from chronic DSS‐induced mice (Figure S3A,B, Supporting Information) unraveled key differences in immune cell infiltration (**Figure** **2**A; Figure S4A, Supporting Information). Comparison of *L. intestinalis* group to control in DSS‐induced colitis model revealed an obvious shift in RORγt^+^ Th17 cells (Figure 2A). ELISA confirmed a significant reduction of Th17‐type cytokine IL‐17A in the *L. intestinalis* group (Figure 2B). Moreover, the expression of genes related to Th17 cells (*Rorc*, *Il17a*, *Il23r*, and *Il21*) and IL‐17 family signaling pathways (*Traf3ip2*) were all decreased in the *L. intestinalis* group (Figure 2C). Meanwhile, no significant shift was found in γδTCR^+^RORγt^+^ T cells (γδT17), the other RORγt^+^ cell populations that secrete IL‐17A (Figure S4B, Supporting Information). Regulatory T cell (Treg) is considered to be closely related to Th17.^[^ ^5^ ^]^ While there was no significant shift in Treg cells (Figure 2A). Concordant with this finding, *L. intestinalis* also reduced Th17 cell response in acute murine colitis (Figure S4C,D, Supporting Information).

We then analyzed the effect of *L. intestinalis* on DSS‐induced colitis in *Rag1* ^−/−^ mice lacking T cells and mice treated with IL‐17A neutralizing antibodies (αIL‐17A). As expected, no significant change in DSS‐treated colitis by *L. intestinalis* was detected in *Rag1* ^−/−^ mice (Figure 2D–F; Figure S4E, Supporting Information). Administration of αIL‐17A (Figure S4F, Supporting Information) also abrogated the protective effect of *L. intestinalis* on colitis (Figure 2G–I; Figure S4G, Supporting Information). These results suggested that *L. intestinalis* suppresses colonic inflammation in a Th17‐cell‐dependent manner.

### 2.3 *L. Intestinalis* Downregulates Serum Amyloid A to Suppress Th17 in Inflammatory Colon

**Figure 3:**
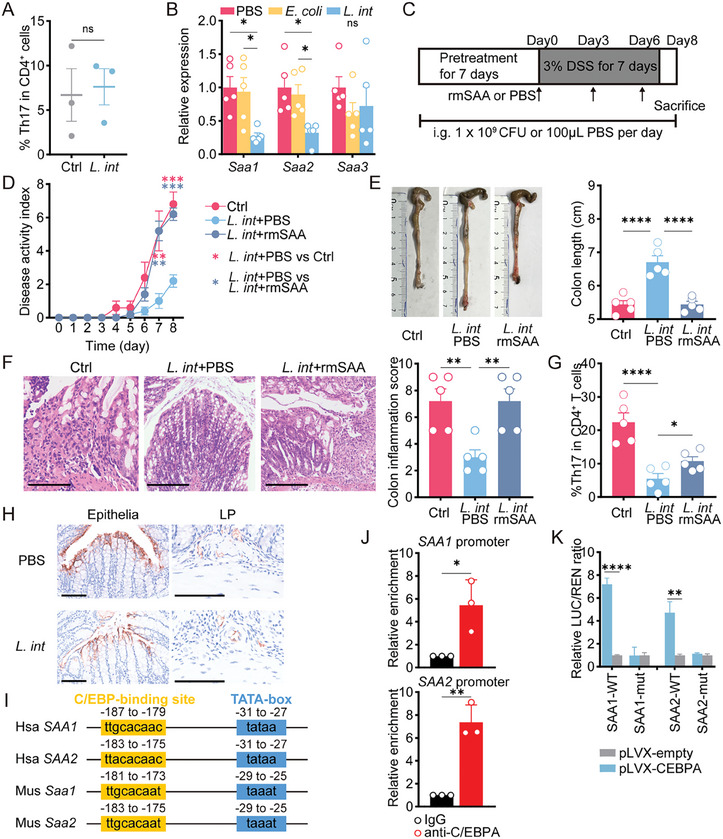
*L. intestinalis* suppressed Th17 cell differentiation by targeting epithelial SAA production. A) In vitro Th17 differentiation assay was performed on naïve CD4^+^ T cells isolated from mouse spleen and treated with PBS (Ctrl) or *L. intestinalis* (*L. int*) (*n* = 3). B) Expression of *Saa1/2/3* was evaluated in bulk colon samples from chronic DSS‐treated mice with gavage of PBS, *E. coli*, or *L. intestinalis* (*L. int*) (*n* = 5). C) Pretreatment with gavage of PBS or *L. intestinalis* (*L. int*) was started on the 7th day before DSS treatment and until the end of the experiment. On days zero, three, and six of DSS course, all mice were intraperitoneally injected (i.p.) with recombinant mouse SAA proteins (rmSAA) or PBS. D–G) The pathology of colitis was evaluated among PBS‐i.p. mice with PBS(PBS+PBS) or *L. intestinalis* (*L. int*+PBS) gavage, as well as rmSAA‐i.p. mice with *L. intestinalis* (*L. int*+rmSAA) gavage, by disease activity index (D), colon length (E), pathological scores (scale bar, 200 µm) (F), and frequencies of Th17 cells in colon LP (G) (*n* = 5). H) Representative fluorescent immunohistochemistry images indicated the expression of *Saa1/2* in colon epithelia and LP from chronic DSS‐treated mice with gavage of PBS and *L. intestinalis* (*L. int*). I) The C/EBP‐binding motifs were conservative among the sequences of promoter regions from human (Hsa) *SAA1/2* genes or mouse (Mus) *Saa1/2* genes. J) The enrichment at SAA1 and SAA2 promoter was detected by ChIP‐qPCR in C/EBPA‐overexpressing HEK‐293T cells using anti‐C/EBPA or control IgG. K) The firefly luciferase (LUC) was driven by wildtype (WT) promoter or mutate (mut) regions of SAA1 and SAA2. The trans‐activation ability of C/EBPA was measured by the LUC / REN ratio in HEK‐293T cells co‐transfected with pLVX‐empty or pLVX‐*CEBPA*. Error bars indicate mean ± SEM. ns, no significance; ^*^ *p* < 0.05; ^**^ *p* < 0.01; ^****^ *p* < 0.0001. *p* values were based on Student's *t*‐test and one‐way ANOVA with post‐hoc test.

Next, we sought to investigate how *L. intestinalis* influenced Th17 cells in the colon. In vitro differentiation of naïve CD4^+^ T cells showed that *L. intestinalis* did not restrain Th17 cell development (**Figure** **3**A), indicating the involvement of alternative pathways rather than a direct effect of *L. intestinalis* on Th17 cells.

Local secretion of serum amyloid A proteins (SAA) has been reported to activate Th17 cells in inflammatory diseases.^[^ ^23^ ^]^ In DSS‐treated mice, gavage of *L. intestinalis* significantly downregulated the colonic expression of *Saa1* and *Saa2*, but not *Saa3* (Figure 3B; Figure S5A, Supporting Information). To validate the role of SAA downregulation in the effect of *L. intestinalis*, we administrated recombinant mouse SAA proteins (rmSAA) in DSS‐treated mice (Figure 3C). Intraperitoneal injection of rmSAA reversed the beneficial effect of *L. intestinalis* in DSS‐treated mice, as indicated by higher disease activity index and more severe inflammation (Figure 3D–F). rmSAA also abolished the reduction of Th17 cell populations by *L. intestinalis* (Figure 3G). These data confirmed the role of SAA downregulation in the protective effect of *L. intestinalis* against colitis.

Other pathways, such as cytokine networks have also been reported to modulate colonic Th17 cells.^[^ ^24^ ^]^ For instance, dendritic cells, macrophages, and their cytokines support Th17 cell differentiation.^[^ ^25^ ^]^ As shown in Figure S4A, dendritic cells and macrophages were not changed by *L. intestinalis*. IL‐23 signaling is involved in the induction of Th17 cells by several gut bacterial species.^[^ ^26^ ^]^ Our results showed that *L. intestinalis* did not suppress the expression levels of *Il23a* (*IL23 p19*) and other cytokines promoting Th17 cells (Figure S5B, Supporting Information). Taken together, these results suggested that *L. intestinalis* inhibited Th17 differentiation by suppressing SAA1 and SAA2.

### 2.4 *L. Intestinalis* Regulates Intestinal Epithelial Production of SAA Proteins in a C/EBPA‐Dependent Manner

Both IECs and macrophages produce SAA locally.^[^ ^27^, ^28^ ^]^ To ascertain the source of SAA‐secreting cells targeted by *L. intestinalis*, we detected SAA expression in the colonic epithelium and LP fractions of DSS‐treated mice with or without *L. intestinalis*. SAA1 and SAA2 were mainly expressed in colonic epithelium of DSS‐treated mice, but not in LP (Figure 3H). *L. intestinalis* mainly reduced expression of *Saa1* and *Saa2* in epithelial cells while not in the LP fractions (Figure 3H; Figure S5C, Supporting Information). We thus identified IECs as the target of SAA reduction by *L. intestinalis*.

To identify the transcription factor that regulates the expression of both SAA1 and SAA2, we used JASPAR and Animal TFDB databases to predict zinc finger protein 384 (ZNF384) and C/EBPA as candidates for further analysis (Figure S5D, Supporting Information). In colon samples of chronic or acute colitis mice models, only *Cebpa* expression was altered by *L. intestinalis*, whereas *Znf384* was unchanged (Figure S5E,F, Supporting Information). The motif prediction also indicated that C/EBP‐binding motifs were relatively conservative among human and mouse SAA1 and SAA2 genes (Figure 3I). To validate the transcriptional regulation of SAA1 and SAA2 by C/EBPA, we examined C/EBPA ChIP‐seq data using CistromeDB, finding C/EBPA bound to the promoter regions of mouse and human *SAA1* and *SAA2* genes (Figure S5G,H, Supporting Information). C/EBPA was overexpressed in HEK‐293T cells, which lacked endogenous C/EBPA,^[^ ^29^ ^]^ (Figure S5I, Supporting Information). In C/EBPA‐overexpressing HEK‐293T, we confirmed the binding of C/EBPA to the promoter of *SAA1* and *SAA2* (Figure 3J). A similar result was found in HT29 cells, a human intestinal epithelial cell line expressing C/EBPA endogenously (Figure S5J, Supporting Information). SAA1 or SAA2 promoter‐driven luciferase reporter system was thus constructed, and dual‐luciferase assays showed that the activity of the two reporters was markedly induced by C/EBPA (Figure S5K‐S5L). Whereas no changes in luciferase activity were induced by C/EBPA in cells transfected with plasmids with mutant predicted binding sites of C/EBPA (**Figure** **5**K). These results validated the involvement of C/EBPA in SAA1 or SAA2 regulation. Collectively, our results suggested that *L. intestinalis* downregulated colonic SAA1 and SAA2 via C/EBPA.

### 2.5 *L. Intestinalis* Increases Retinoic Acid Production and Host Biosynthesis to Attenuate Colitis

**Figure 4:**
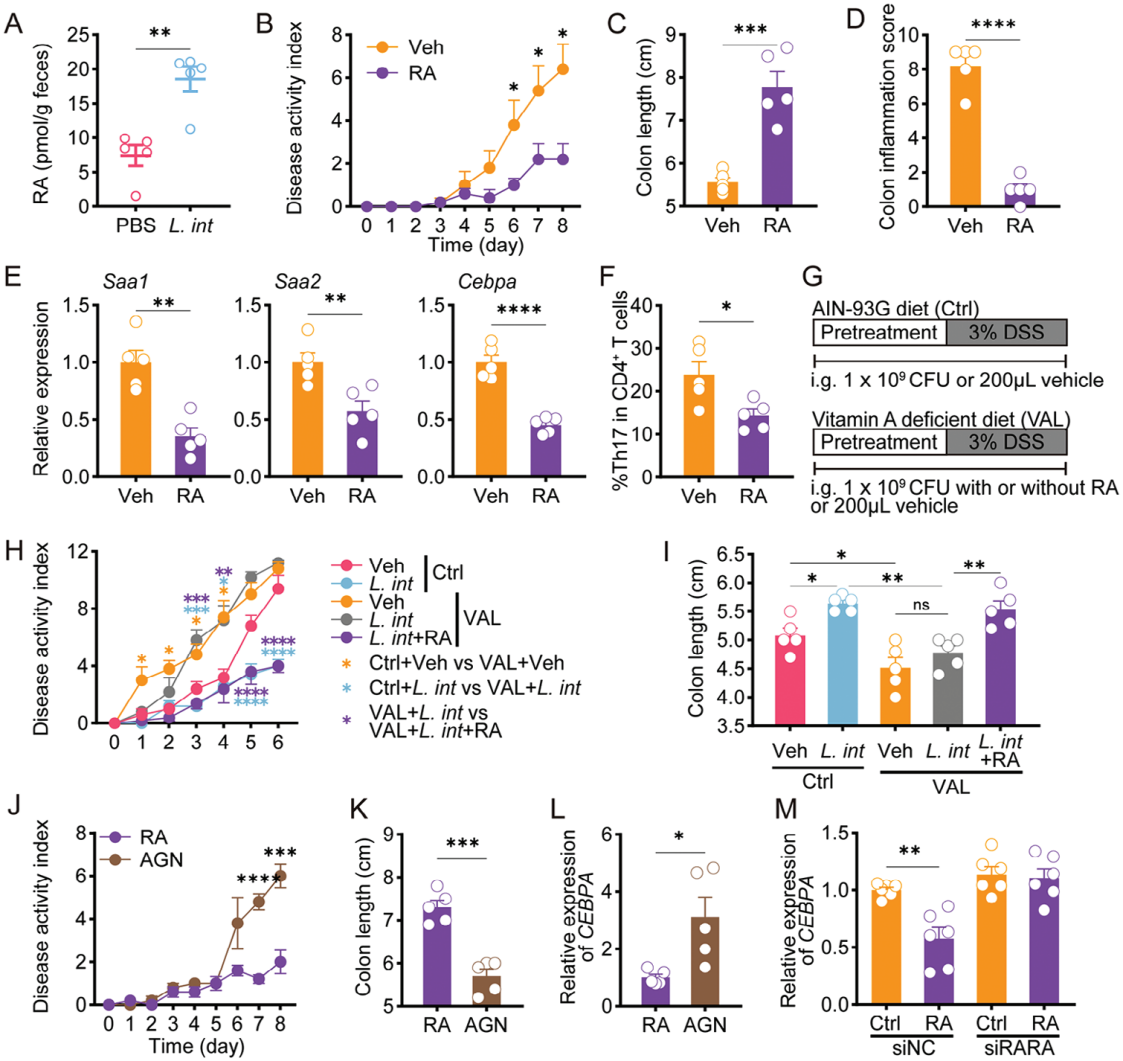
*L. intestinalis*‐associated RA reduced C/EBPA‐driven SAA production in RAR‐α‐mediated manner. A) Quantification of RA was performed in the feces of acute DSS‐treated mice with PBS or *L. intestinalis* (*L. int*) gavage (*n* = 5). B–F), The pathology of acute colitis and related gene expressions were evaluated between DSS‐treated mice with vehicle (Veh) or RA gavage, by disease activity index (B), colon length (C), pathological scores (D), colonic expression of *Saa1*, *Saa2*, and *Cebpa* (E), and frequencies of Th17 cells (F) (*n* = 5). G) 2 groups were fed with AIN‐93G as a control diet (Ctrl). Three groups were fed with a vitamin A‐deficient diet (VAL). Pretreatment with gavage of PBS, *L. int*, or *L. int*+RA was started on the 7th day before DSS treatment and until the end of the experiment. All mice received a 6‐day 3% DSS treatment. H,I) The pathology of acute colitis was evaluated among Ctrl or VAL‐fed mice with respective gavage, by disease activity index (H) and colon length (I) (*n* = 5). J–L), The pathology of acute colitis and related gene expressions were evaluated between DSS‐treated mice with vehicle RA or RA+AGN193109 (AGN) administration, by disease activity index (J), colon length (K), and *Cebpa expression* (L) (*n* = 5). M) In HT29 cells treated by siRARA, the effect of RA on the expression of C/EBPA was analyzed. Error bars indicate mean ± SEM. ns, no significance; ^*^ *p* < 0.05; ^**^ *p* < 0.01; ^***^ *p* < 0.001; ^****^ *p* < 0.0001. *p* values were based on Student's t‐test and one‐way ANOVA with post‐hoc test.

*L. intestinalis* was reported to restore vitamin A metabolism and induce RA biosynthesis in antibiotic‐treated mice.^[^ ^19^ ^]^ RA is a key derivative of vitamin A, which has been considered to relieve colitis.^[^ ^16^ ^]^ We found that *L. intestinalis* treatment elevated fecal RA levels in colitis mice, as well as in culture media inoculated with *L. intestinalis* (**Figure** **4**A; Figure S6A, Supporting Information). In addition, RA supplementation exerted an anti‐colitis effect (Figure 4B–D; Figure S6B–E, Supporting Information). Similar to *L. intestinalis*, RA inhibited the expression of *Saa1*, *Saa2*, and *Cebpa*, and influenced Th17 cell differentiation (Figure 4E,F). We then fed mice with AIN‐93G control diet, or vitamin A‐deficient diet to lower vitamin A content in colonic lumen (VAL) (Figure 4G). In VAL mice, *L. intestinalis* gavage did not relieve colitis (Figure 4H,I). However, the additional RA rescued anti‐colitis effect of *L. intestinalis* in VAL mice (Figure 4H,I).

RA exerts its bioactivity predominantly by binding to retinoic acid receptors (RARs), which function as transcription factors or display non‐genomic effects.^[^ ^30^ ^]^ We treated mice with AGN193109 (AGN), a pan‐RARs antagonist, to block the function of RARs. Although receiving RA administration, AGN‐treated mice developed more severe colitis with more Th17 cell infiltration and higher epithelial expression of *Saa1*, *Saa2*, and *Cebpa* (Figure 4J–L; Figure S6F,G, Supporting Information), suggesting that RA could regulate C/EBPA in colitis via RARs. In HT29 cells, RA also reduced C/EBPA expression through RARs (Figure S6H, Supporting Information). The effect of RA on SAA promoter‐driven luciferase activity differed in HT29 cells (endogenously expressing C/EBPA) and HEK‐293T cells (lacking endogenous C/EBPA) (Figure S6I, Supporting Information), suggesting that C/EBPA was involved in RA‐induced change in SAA expression. Bioinformatic analysis suggested that RARα had higher regulatory potential scores, and was a potential regulatory element of C/EBPA (Figure S6J, Supporting Information). Knockdown of RARα abrogated the effect of RA (Figure 4M; Figure S6K, Supporting Information), demonstrating that RARα mediated the regulation of C/EBPA by RA. We identified a potential RARα‐binding site in the +9‐kb region of *CEBPA* gene, a tissue‐broad regulatory site of C/EBPA,^[^ ^31^ ^]^ (Figure S6L, Supporting Information), and further confirmed the binding of RARα to this site by ChIP‐qPCR assay (Figure S6M, Supporting Information). Whereas, no possible RARα binding site was present in the 5′ flanking region of C/EBPA, nor in the flanking regions of SAA1 or SAA2 (Figure S6L, Supporting Information). These data suggested that RA treatment might trigger gene expression cascades – RA decreased C/EBPA expression via RARα, and the decrease in C/EBPA could lead to a reduction of SAA1 and SAA2.

**Figure 5:**
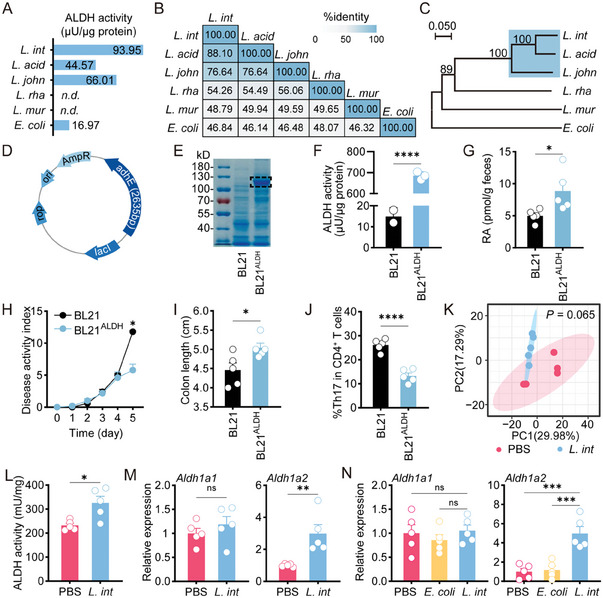
*L. intestinalis* promoted RA synthesis through its own ALDH and by enhancing host ALDH. A) The ALDH activity was detected in six different species. B) Percentage identity analysis was performed on ALDH protein sequences of six different species. C) Phylogenetic tree analysis of ALDH protein sequences of six different species was constructed. Bootstrap analysis values for 1000 replicates were shown. D–F), Plasmid containg *L. intestinalis* adhE (D) was transduced into bacterial expression systems BL21^ALDH^. The ALDH expression and activity were detected (E–F). G–J), Quantification of RA was performed in the feces of acute DSS‐treated mice with BL21 or BL21^ALDH^ gavage (G). The pathology of acute colitis was evaluated between DSS‐treated mice with BL21 or BL21^ALDH^ gavage, by disease activity index (H) and colon length (I), and frequencies of Th17 cells (J) (*n* = 5). K) β‐diversity of the fecal was compared between chronic DSS‐treated mice with PBS gavage and with *L. intestinalis* (*L. int*) gavage (*n* = 5). L,M) The ALDH activity (L) and *related* gene expression (M) were detected between colon tissues of DSS‐induced acute colitis mice with PBS, and *L. intestinalis* (*L. int*) gavage (*n* = 5). N) Expression levels of *Aldh1a1* and *Aldh1a2* were evaluated in bulk colon samples from chronic DSS‐treated mice with gavage of PBS, *E. coli*, and *L. intestinalis* (*L. int*) (*n* = 5). Error bars indicate mean ± SEM. ns, no significance; ^*^ *p* < 0.05; ^**^ *p* < 0.01; ^***^ *p* < 0.001. *p* values were based on Student's t‐test, PERMANOVA test, and one‐way ANOVA with post‐hoc test.

ALDH, a key enzyme for RA synthesis, is expressed in *L. intestinalis*.^[^ ^32^ ^]^ Since the genes encoding ALDH are widely represented in prokaryotes, we compared ALDH activity between *L. intestinalis* and other gut commensals. We thus selected *E. coli* and several species belonging to *Lactobacillus* genus, such as *L. acidophilus*, *L. johnsonii*, *L. rhamnosus* and *L. murinus*. Except for *L. rhamnosus* and *L. murinus*, the other species tested all possessed ALDH activity, among which *L. intestinalis* had the highest activity (Figure 5A). We then compared the amino acid sequence of their ALDH. Percent identity analysis revealed higher identity among *L. intestinalis*, *L. acidophilus*, and *L. johnsonii*, but not with *L. rhamnosus* and *L. murinus* (≈50) lacking in ALDH activity (Figure 5B). Phylogenetic analysis based on ALDH sequences revealed that *L. intestinalis* clustered together with *L. acidophilus* and *L. johnsonii* but had evolved away from *L. rhamnosus* and *L. murinus* (Figure 5C). These in silico results illustrated the differences in ALDH activity among the members of *Lactobacillus* genus and showed that *L. intestinalis* is unique in having high ALDH activity. We expressed *L. intestinalis* ALDH (encoded by gene adhE) in BL21 (BL21^ALDH^) (Figure 5D,E). Compared with wildtype BL21, BL21^ALDH^ possessed higher ALDH activity and produced more RA, which improved colitis and decreased the frequency of Th17 cells (Figure 5F–J).

In addition to their own metabolic activities, gut commensals affect vitamin A turnover by regulating gut microbiome or altering host enzymes.^[^ ^33^ ^]^ 16S rRNA sequencing revealed no significant differences in the bacterial community richness and diversity (α‐diversity) after *L. intestinalis* gavage (Figure S7A, Supporting Information). *L. intestinalis* supplementation had no impact at the genus level (Figure S5B, Supporting Information). The principal co‐ordinates analysis of OTUs data (β‐diversity) showed no significant difference between *L. intestinalis* and PBS controls (Figure 5K). We then investigated the impact of *L. intestinalis* on host enzymes. *L. intestinalis* increased ALDH activity in the colon of acute DSS‐treated mice (Figure 5L). By analyzing the expression of ALDH family in mice, we demonstrated that *L. intestinalis* induced higher colonic expression of *Aldh1a2* (Figure 5M). Similar results were observed in chronic DSS‐treated mice (Figure 5N). Taken together, we found that RA biosynthesis was required for the protective effect of *L. intestinalis*, and both the metabolic activity of *L. intestinalis* and induction of host RA metabolism by *L. intestinalis* contributed to the elevated RA levels in vivo.

### 2.6 *L. Intestinalis* has Potential Therapeutic Effects on Ulcerative Colitis

**Figure 6:**
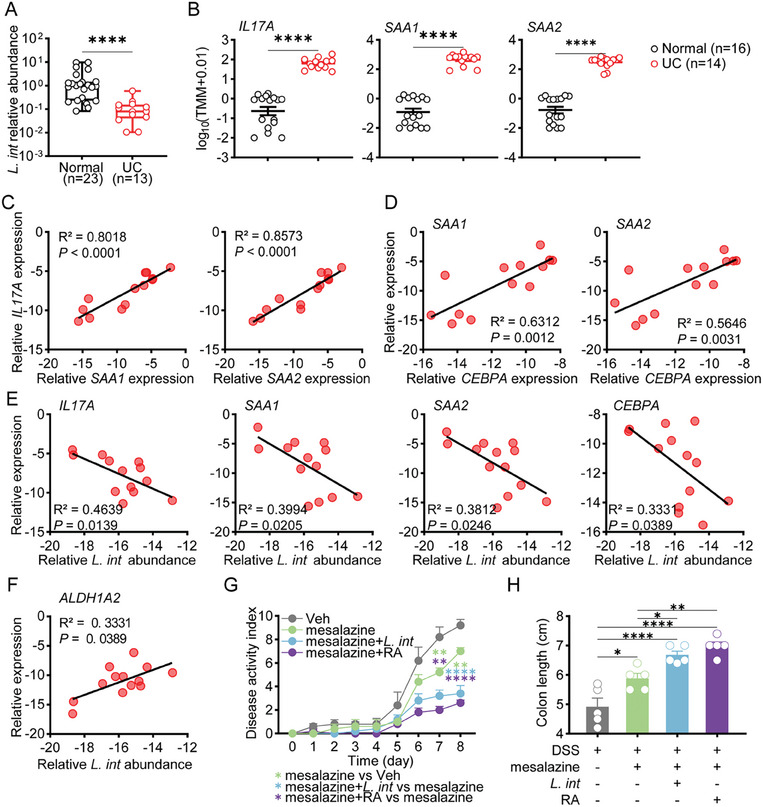
*L. intestinalis* suppressed the C/EBPA‐SAA1/2‐Th17 axis in UC patients and exerted therapeutic effect on DSS‐induced colitis. A) Relative abundance of *L. intestinalis* was compared between normal colon samples and UC patient inflammatory samples. B) Colonic expression of *IL17A*, *SAA1*, and *SAA2* was compared between UC patients and healthy people from GEO data (GSE128682). C–F) The correlation analysis was performed in colon samples from UC patients to determine the relationship between the expression of *IL17A* and *SAA1/2* (C), between expression of *SAA1/2* and *CEBPA* (D), and between expression of genes (*IL17A*, *SAA1/2*, *CEBPA*, *ALDH1A2*) and *L. intestinalis* abundance (*L. int*) (E–F) (*n* = 13). G,H) The pathology of acute colitis was evaluated among DSS‐treated mice treated with vehicle (Veh), or mesalazine alone or in combination with *L. intestinalis* (*L. int*) or RA, by disease activity index (G) and colon length (H) (*n* = 5). Error bars indicate mean ± SEM. ^*^ *p* < 0.05; ^**^ *p* < 0.01; ^***^ *p* < 0.001; ^****^ *p* < 0.0001. *p* values were based on Mann–Whitney test, Student's *t*‐test, one‐way ANOVA with post‐hoc test, and Pearson correlation test.

We further evaluated the clinical significance of *L. intestinalis* in UC patients. qPCR of colonic tissues showed a remarkable reduction of *L. intestinalis* in UC donors as compared to healthy controls (**Figure** **6**A). On the contrary, expression of *IL17A*, *SAA1*, and *SAA2* was upregulated in UC patients (Figure 6B) in a previously published mRNA‐Seq dataset.^[^ ^34^ ^]^

The correlation between *L. intestinalis* and C/EBPA‐SAA1/2‐IL17A axis was next analyzed in colon samples. The expression *of IL17A, CEBPA* were positively correlated with *SAA1* and *SAA2* (Figure 6C,D). In contrast, the higher abundance of *L. intestinalis* corresponded to lower expression of *IL17A*, *SAA1*, *SAA2*, and *CEBPA* (Figure 6E).

In human feces, we found that the higher abundance of *L. intestinalis* was associated with higher fecal RA levels (Figure S8A, Supporting Information). In vitro fermentation system also confirmed that *L. intestinalis* converted vitamin A to RA in the background of human microbiome (Figure S8B, Supporting Information). The expression of human *ALDH1A2*, orthologous to murine *Aldh1a2*, was also associated with *L. intestinalis* abundance (Figure 6F). Collectively, these results implied that *L. intestinalis* increased RA synthesis in human samples and suppressed C/EBPA‐SAA1/2‐IL17A axis.

Mesalazine is the first‐line treatment for UC patients. The combination therapy of *L. intestinalis* or RA with mesalazine was evaluated in DSS‐challenged mice. The combination of mesalazine and *L. intestinalis* or RA displayed a better therapeutic effect in terms of disease activity, colon length, and spleen weight (Figure 6G,H; Figure S8C,D, Supporting Information). *L. intestinalis* and associated RA metabolites have significant therapeutic effects in colitis.

## 3 Discussion

The intestinal immune system involves the interplay of adaptive and innate immunity, and epithelial barrier function.^[^ ^35^ ^]^ Despite the close interactions between gut microbiota and host immune response, the specific effects of different microorganisms are not well understood. Here, we demonstrated that the colonization of *L. intestinalis* attenuated colitis severity, maintained intestinal integrity, and limited Th17 cell differentiation. We confirmed that *L. intestinalis* abundance declined in UC patients and was related to *IL17A* expression.

Th17 cells, a subpopulation of CD4^+^ T cells, secrete IL‐17 with the activation of RORγt, and participate in protection against intestinal pathogens. In the context of colitis, Th17 cells and their associated cytokines exert proinflammatory effects to drive inflammation and damage.^[^ ^36^ ^]^ The modulatory effects of microbes on intestinal resident Th17 cells have also been reported. For example, enterotoxigenic *Bacteroides fragilis* promotes Th17 cells by altering exosomal miRNA derived from host cells,^[^ ^37^ ^]^ whereas *Eggerthella lenta* activates Th17 cells by metabolizing immunomodulatory compounds.^[^ ^6^ ^]^ Meanwhile, several probiotics have been shown to alter Th17 response to suppress colon inflammation,^[^ ^38^ ^]^ but their underlying mechanism remain poorly understood.

Our results suggested that *L. intestinalis* did not directly inhibit Th17 differentiation but required the blockade of IECs‐dependent SAA production. Due to structural and functional divergence, the different isoforms of SAA play distinctive roles in health and disease.^[^ ^9^, ^39^ ^]^ For example, in murine experimental autoimmune encephalomyelitis, hepatic SAA1 and SAA2 are first activated to prime systemic response, followed by an increase in SAA3 of central nervous system.^[^ ^23^ ^]^ In our model, *L. intestinalis*‐mediated anti‐inflammation primarily involves downregulation of SAA1 and SAA2 but not SAA3. In agreement with our observations, Medina‐Rodriguez et al.^[^ ^40^ ^]^ reported that SAA1 and SAA2 are functional SAA proteins mediating the interaction between gut microflora and host intestine.^[^ ^40^ ^]^ Although multiple transcription factors are involved in intrahepatic SAA regulation, the regulation of extrahepatic SAA is largely unknown. Here, we validated the transcriptional regulation of SAA1 and SAA2 by C/EBPA, and elucidated that colonization with *L. intestinalis* reduced the expression of C/EBPA in colon epithelial cells. C/EBP family are known to regulate hematopoiesis and immunity.^[^ ^41^ ^]^ Concordantly, small IECs were shown to express increased SAA1 with C/EBPD activation,^[^ ^36^ ^]^ supporting that C/EBP family is involved in the extrahepatic production of SAA.

The gut microbiota is known to expand metabolic capabilities of the host, which can serve critical roles in health and disease. RA, an important derivative of vitamin A metabolism, has a therapeutic effect on UC according to several clinical and preclinical studies.^[^ ^16^, ^17^ ^]^ Here, we showed that *L. intestinalis* not only metabolized vitamin A via its intrinsically high ALDH activity, but also enhanced RA metabolism in the host intestine. Hence, co‐metabolism of *L. intestinalis* and the host contributes to increased RA biosynthesis in vivo, and *L. intestinalis* is a safe and effective probiotic in boosting the beneficial effect of vitamin A in UC treatment.

RA is a ligand binding to RARs, which activate or repress transcription of numerous genes important for physiological events.^[^ ^42^ ^]^ In addition to suppressing inflammation in autoimmunity.^[^ ^43^, ^44^ ^]^ RA exhibits more comprehensive benefits, such as enhancing defense responses.^[^ ^45^ ^]^ Our findings suggested that in colitis, RA mediated the protective effect of *L. intestinalis* by altering the C/EBPA‐SAA1/2‐Th17 axis. RA‐induced SAA downregulation has also been reported in colitis.^[^ ^46^ ^]^ However, according to Gattu et al.^[^ ^47^ ^]^ IECs‐specific knockout of RARβ (a kind of RARs isoform) leads to extremely low SAA expression in small intestines,^[^ ^47^ ^]^ indicating that RARβ is required for SAA expression of small IECs. Intriguingly, our results enriched the involvement of RARβ in the regulation of SAA expression, suggesting that short‐term RARs‐pan‐antagonist treatment blocked RA‐induced downregulation of SAA1/2 with crosstalk to C/EBPA in colitis colonic epithelial cells. Given the comprehensive role of RA, we propose that the effects of RA vary depending on the pathologic circumstances (different cytokine networks or receptors), the targeted cells and tissues, as well as the duration of treatment.

In conclusion, we identified that *L. intestinalis* increased colon RA metabolism, which relayed its signals by suppressing SAA1/2 production, and consequently restraining Th17 cells to alleviate experimental colitis. Our work shed further light into the interplay between the host and gut microbes in the pathogenesis of UC and suggested that *L. intestinalis* was a promising probiotic in alleviating UC.

## 4 Experimental Section

### Animal Models

All mice were housed at Sir Run Run Shaw Hospital animal facility under specific pathogen‐free (SPF) conditions, with a 12‐h light/dark cycle. Experimental procedures were conducted in accordance with the guidelines of the Institutional Animal Use. All experimental protocols were approved by the Animal Experimentation Ethics Committee at Sir Run Run Shaw Hospital, School of Medicine, Zhejiang University (NO. SRRSH202107028; NO. ZJU20220379). Animals were assigned randomly to experimental groups. Before bacterial intragastric administration, mice received a 3‐day antibiotic treatment. During intragastric administration, live *L. intestinalis*, live *E. coli* MG1655, or 200 µL PBS were administrated. The colonization of *L. intestinalis* was detected by quantitative real‐time PCR.

Murine chronic and acute colitis was induced by DSS (160 110, MP Biomedicals).^[^ ^3^ ^]^ For chronic colitis, mice were given with 2% DSS in drinking water for 5 days, followed by 14 days of normal water; and this process was repeated three times. For acute colitis models, mice were pretreated with oral gavage for 7 days, and then were administrated by 7‐day 3% DSS treatment with oral gavage. Disease progression and body weight were monitored daily. The DAI was the sum of weight loss, stool consistency, and rectal bleeding.^[^ ^3^ ^]^

### Bacteria

*L. intestinalis* ATCC 49 335, *L. murinus* ATCC 35 020, *E. coli* MG1655, and *E. coli* BL21 were obtained from Biobw Biotechnology Co., Ltd or Vazyme Biotech Co., Ltd. *L. johnsonii*, *L. acidophilus* NCFM, and *L. rhamnosus* were gifts from Dr. Wei Liu.

### Cell Lines and Cell Culture

The HEK‐293T and HT29 cell lines were cultured in DMEM and McCoy's 5A medium respectively at 37 °C in 5% CO_2_. The culture medium was supplemented with 10% FBS and 1% penicillin/streptomycin.

### Histological Analysis

The tissues were fixed and stained with hematoxylin and eosin (H&E). Two investigators independently assessed the degree of colitis in a blinded fashion by the following criteria: severity of inflammation (0, none; 1, mild; 2, moderate; 3, severe), inflammation infiltration (0, none; 1, mucosa; 2, mucosa and submucosa; 3, transmural) and crypt damage (0, none; 1, basal 1/3; 2, basal 2/3; 3, crypt loss; 4, crypt and surface epithelial destruction).

### Isolation of Colonic Epithelial and LP Samples

Colons were collected and opened longitudinally, were then cut into small pieces to be incubated in D‐Hanks buffer containing 1 mm Dithriothreitol (MB3047‐1, Meilunbio) and 5 mm EDTA (MB2514, Meilunbio) with shaking at 37 °C for 30 min. Epithelial fractions were dissociated from this treated tissue. After epithelial removal, the remaining tissue was then minced into 1–2 mm pieces and was placed in Hanks buffer (MA0041, Meilunbio) containing 5% heat‐inactivated fetal calf serum and 1 mg mL^−1^ Type IV collagenase (A005318, Sangon) for 30 min at 37 °C shaker. The cell suspension was passed through a 200‐mesh filter and then was collected as LP samples.

### Flow Cytometry

Colon LP cells were counted and were stained with Fixable viability stain 510 (564 406, BD Biosciences). After washing in FACS buffer (PBS + 1% BSA), cells were treated with fluorochrome‐conjugated antibodies for surface antigens at 4 °C for 30 min. Next, cells were permeabilized with fixation/permeabilization buffer (eBioscience) and were intracellular stained at room temperature for 40 min.

### Isolation and In Vitro Differentiation of Naïve CD4+ T Cells

Naïve CD4^+^ T cells were isolated using a naïve CD4^+^ T cell isolation kit (19 765, Stemcell). Naïve CD4^+^ T cells were seeded onto a 48‐well plate coated with 1 mg mL^−1^ anti‐CD3 (100 340, Biolegend) and 1 mg mL^−1^ anti‐CD28 (102 122, Biolegend). Cells were stimulated for 72 h with 15 ng mL^−1^ IL‐1β (575 704, Biolegend), 30 ng mL^−1^ IL‐6 (100 340, Biolegend), 60 ng mL^−1^ IL‐23 (589 004, Biolegend), 5 µg mL^−1^ anti‐IL‐4 (504 135, Biolegend) and 15 ng mL^−1^ anti‐IFNγ (505 847, Biolegend). Naïve CD4^+^ T cells were cultured and stimulated in antibiotic‐free IMDM (PM150510, Procell) with 0.1 m β‐mercaptoethanol (PB180633, Procell). *L. intestinalis* was added for coculture with cells at a MOI of 100:1 for last 24 h.

### Qualification of RA in Feces

Accurately weighed feces samples were resuspended with 150 µL of ice‐cold PBS, homogenized and was added with 150 µl of Acetonitrile, vortexed. 900 µL of Methyl‐Tert‐Butyl Ether was added to each tube and vortexed for 30 s and followed by setting at −20°C for 1 h prior to centrifugation for 20 min at 9000 rcf. The upper organic layer was transferred to a new test tube and dried by vacuum. The extract was reconstituted in 1000 µL methanol and centrifugated for further analysis. Prominence LC‐20A with Waters UPLC BEH Amide column (1.7 um; 2.1 ×100 mm Column) coupled to AB SCIEX 5500 QTRAP Q‐Lit mass spectrometer was used for LC‐MS/MS analysis. The mobile phase consisted of water + 5% acetonitrile with 20 mm ammonium acetate (A) and acetonitrile (B) and the flow rate was 0.4 mL mi^−1^n. The initial mobile phase was 90% B held for 1 min before a gradient to 55% B at 5 min then decreased to 40% B from 5 min to 6 min and held for 1.7 min before returning to initial conditions. The MS was operated in positive ion atmospheric pressure chemical ionization (APCI) mode and data collected using multiple reaction monitoring.

### Human Colon Biopsies

Studies were approved by the Medical Ethics Committee of Sir Run Run Shaw Hospital, School of Medicine, Zhejiang University (20211103‐35). We used the sample from a previous cohort, including control individuals (*n* = 23), who rejected diarrhea history or recent treatment of antibiotics or probiotics, as well as UC patients (*n* = 13), who were diagnosed by endoscopy and histopathology without immunotherapy or antibiotic treatment in Sir Run Run Shaw Hospital, School of Medicine, Zhejiang University.

### In Vitro Fermentation System

Feces were obtained from IBD patients. 4–5 samples were pooled in one test. Sterile PBS was used to prepare 10% (w/v) fecal suspension. The basal nutrient medium was described as before.^[^ ^48^ ^]^ Fecal suspension was inoculated into the culture medium with or without *L. intestinalis*, and incubated at 37°C for 24 h in anaerobic chamber. At the end of the incubation, vitamin A was added and incubated with bacteria at 37°C for 3 h in the dark.^[^ ^19^ ^]^ The culture supernatants were collected for measurement of retinoic acid.

### 16S rRNA sequencing

Total microbial genomic DNA was isolated from mouse feces followed by concentration measurement and quality check. PCR was performed using GeneAmp PCR System 9700 (ABI) and primer pairs 338 F and 806 R to the V3–V4 hypervariable regions of the 16S rRNA gene. The purified PCR amplicons were quantified by QuantiFluor‐ST (Promega) and used as template for sequencing by Illumina MiSeq platform (Illumina) according to the protocols of Shanghai MajorBio. Quality‐filter‐passed data were obtained by Trimmomatic and merged by FLASH. UPARSE (http://drive5.com/uparse/) was used to cluster OTUs. Bioinformatic analysis based on rarefied data was performed using the Majorbio Cloud platform (https://cloud.majorbio.com).

### Statistical Analysis

The experimental data was expressed as mean ± standard error of mean (SEM). All statistical analyses were performed using GraphPad Prism 9.0 software (GraphPad Software) or the Majorbio Cloud platform (https://cloud.majorbio.com). Unpaired Student's *t*‐test, one‐way ANOVA test followed by Tukey's post hoc test, Kruskal‐Wallis test, Mann Whitney test, or Wilcoxon matched‐pairs signed‐rank test were used as indicated. Spearman correlation analysis was used for correlation analysis. *p*‐value < 0.05 was considered statistically significant.

## Conflict of Interest

The authors declare no conflict of interest.

## Author Contributions

Q.‐W. W., D. J., J. H., and Y. S. contributed equally to perform most experiments and wrote the original draft. Y. Q., Q.‐W. G., Y.‐D. Q., Q.‐Y. W., Y.‐Y. H., L. W., Y.‐F. F., H.‐Q. H., M. L., L.‐J. F., and J.‐M. S contributed to performing experiments and/or data analysis and interpretation. S.‐J. C., L.‐J. W., and Z.‐F. S. designed and supervised the study, analyzed, and interpreted the data, and wrote and revised the manuscript. All authors read and approved the final manuscript.

## Supporting information



Supporting Information

## Data Availability

The data that support the findings of this study are openly available in JASPAR; AnimalTFDB; and CistromeDB at https://jaspar.genereg.net/; http://bioinfo.life.hust.edu.cn/AnimalTFDB/; http://cistrome.org/db/, reference number 3006, 100475.
