# Supplementary material for: Lactobacillus Intestinalis Primes Epithelial Cells to Suppress Colitis‐Related Th17 Response by Host‐Microbe Retinoic Acid Biosynthesis
Source: Adv Sci (Weinh). 2023 Nov 20;10(36):2303457. doi: 10.1002/advs.202303457 (PMC10754072; doi:10.1002/advs.202303457)
Supplement: Supplementary file 1 — Supporting Information [file ADVS-10-2303457-s001.pdf]

ADVANCED SCIENCE
Open Access

Supporting Information

for *Adv. Sci.*, DOI 10.1002/advs.202303457

Lactobacillus Intestinalis Primes Epithelial Cells to Suppress Colitis-Related Th17 Response by Host-Microbe Retinoic Acid Biosynthesis

Qi-Wen Wang, Ding-Jia-Cheng Jia, Jia-Min He, Yong Sun, Yun Qian, Qi-Wei Ge, Ya-Dong Qi, Qing-Yi Wang, Ying-Ying Hu, Lan Wang, Yan-Fei Fang, Hui-Qin He, Man Luo, Li-Jun Feng, Jian-Min Si, Zhang-Fa Song, Liang-Jing Wang* and Shu-Jie Chen**

Supporting Information

L. intestinalis **primes epithelial cells to suppress colitis-related Th17 response by host-microbe retinoic acid biosynthesis**

Qi-Wen Wang, Ding-Jia-Cheng Jia, Jia-Min He, Yong Sun, Yun Qian, Qi-Wei Ge, Ya-Dong Qi, Qing-Yi Wang, Ying-Ying Hu, Lan Wang, Yan-Fei Fang, Hui-Qin He, Man Luo, Li-Jun Feng, Jian-Min Si, Zhang-Fa Song, Liang-Jing Wang, Shu-Jie Chen

Figure S1, related to Figure1

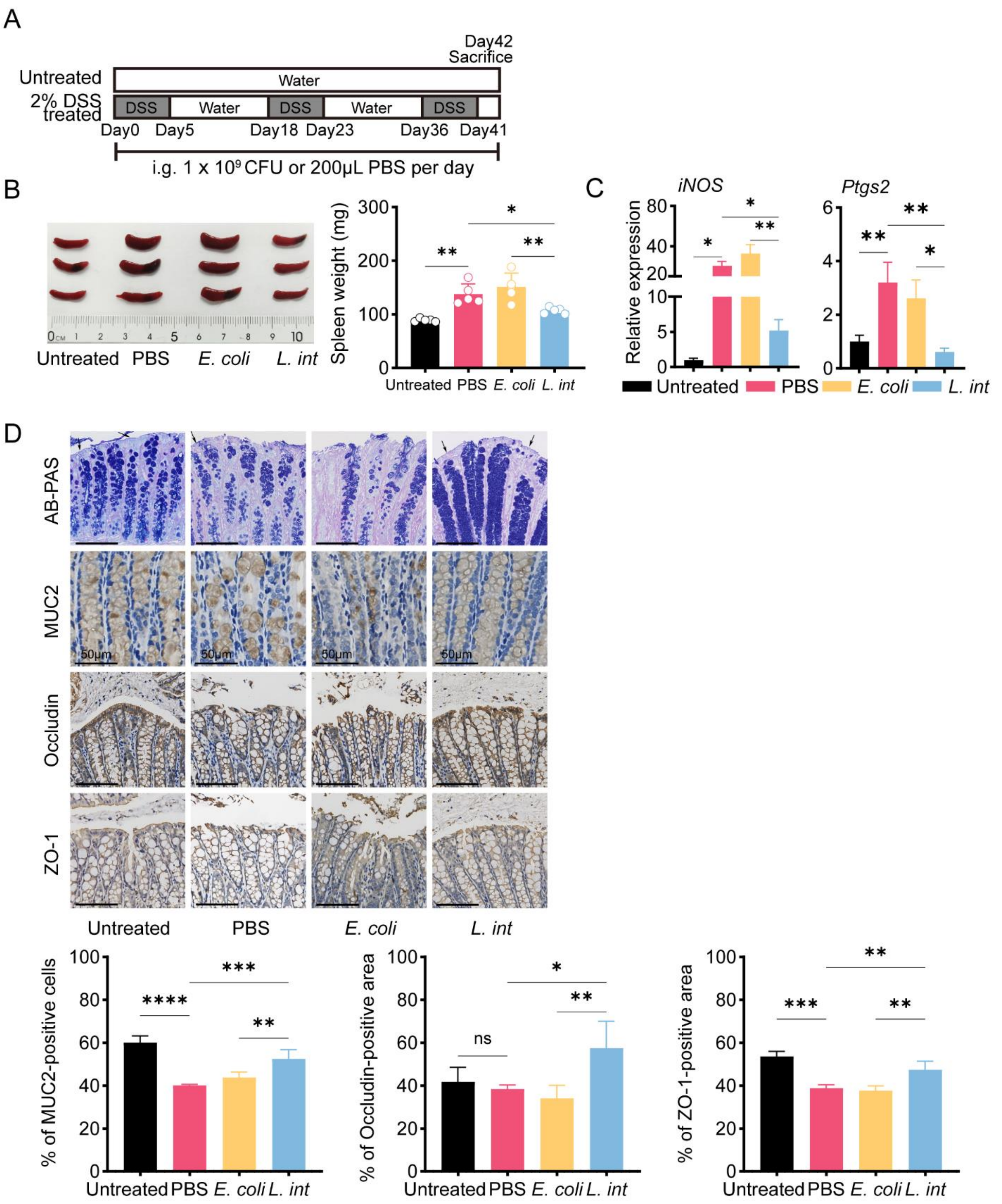

Figure S1, related to Figure 1. ***L. intestinalis*** **relieved DSS-induced colitis**

A, Chronic colitis group mice were treated with 3 cycles of 5-day 2% DSS in drinking water followed by 14-day normal water. Control group mice received normal water during the experiment. **B-D,** spleen size and weight **(B)**, and colonic expression of *iNos* and *Ptgs2* **(C)** were compared among mice treated without (Untreated), or with DSS accompanied by PBS, *E. coli*, or *L. intestinalis* (*L. int*) gavage respectively (n = 5). Alcian blue-PAS staining (scale bar, 100 μm), and the MUC2 (scale bar, 50 μm), Occludin (scale bar, 100 μm) and ZO-1 (scale bar, 100 μm) immunohistochemistry were used for assessment of mucosal barrier **(D)** (n = 3). Error bars indicate mean ± SEM. *P < .05; **P < .01; ***P < .001; ****P < .0001. P values were based on Mann-Whitney test, Kruskal-Wallis with post-hoc test, and one-way ANOVA with post-hoc test.

Figure S2, related to Figure1

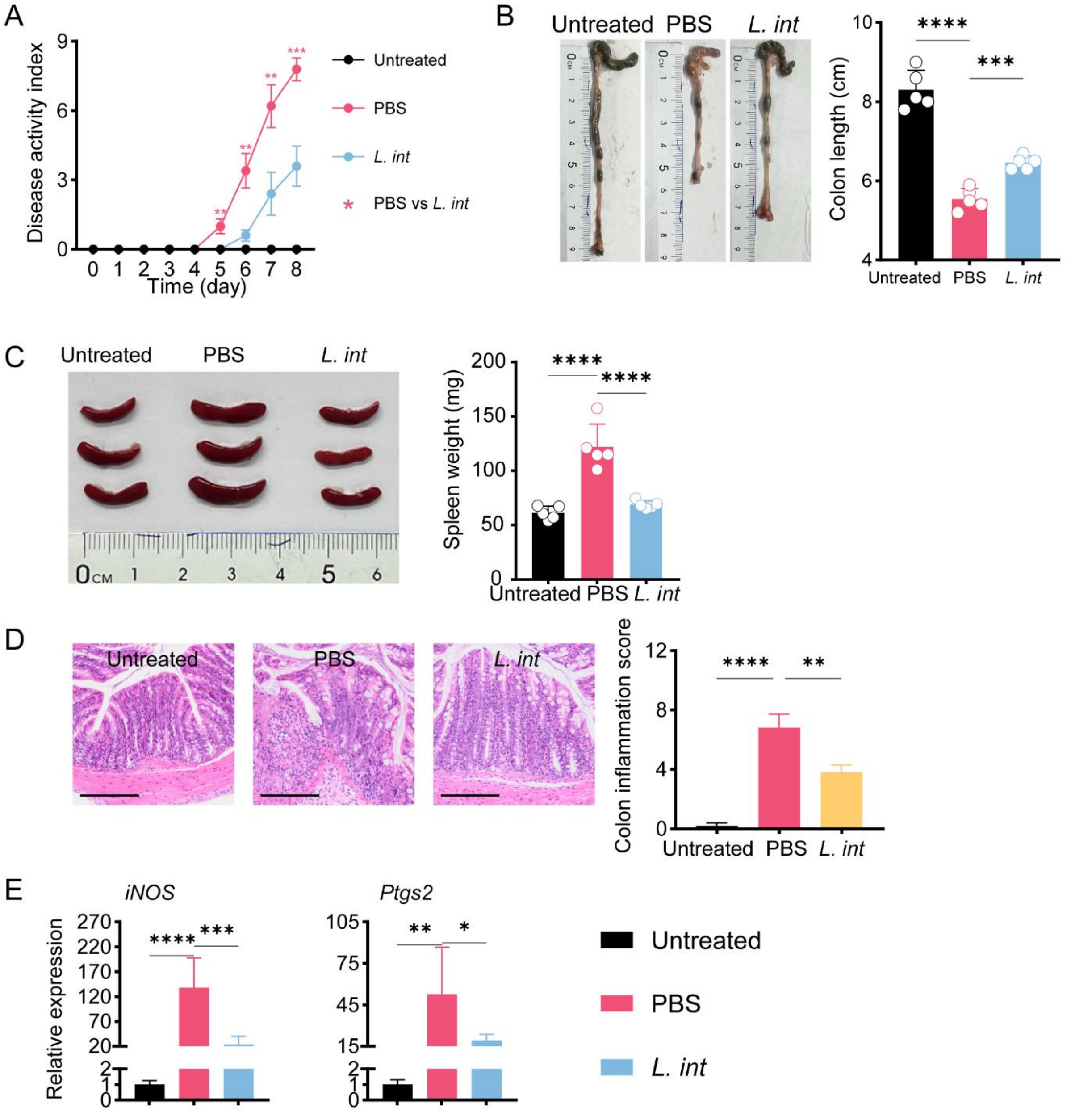

Figure S2, related to Figure 1. ***L. intestinalis*** **relieved DSS-induced acute colitis**

A-E, The pathology of colitis and systemic inflammation was evaluated among control group (Untreated) and the 2 chronic colitis groups with PBS, and *L. intestinalis* (*L. int*) gavage respectively by disease activity index **(A)**, colon length **(B)**, spleen size and weight **(C)**, histological score (scale bar, 200 μm) **(D)**, and colonic expression of *iNos* and *Ptgs2* **(E)** (n = 5). Error bars indicate mean ± SEM. *$P < .05$; **$P < .01$; ***$P < .001$; ****$P < .0001$. *P* values were based on one-way ANOVA with post-hoc test.

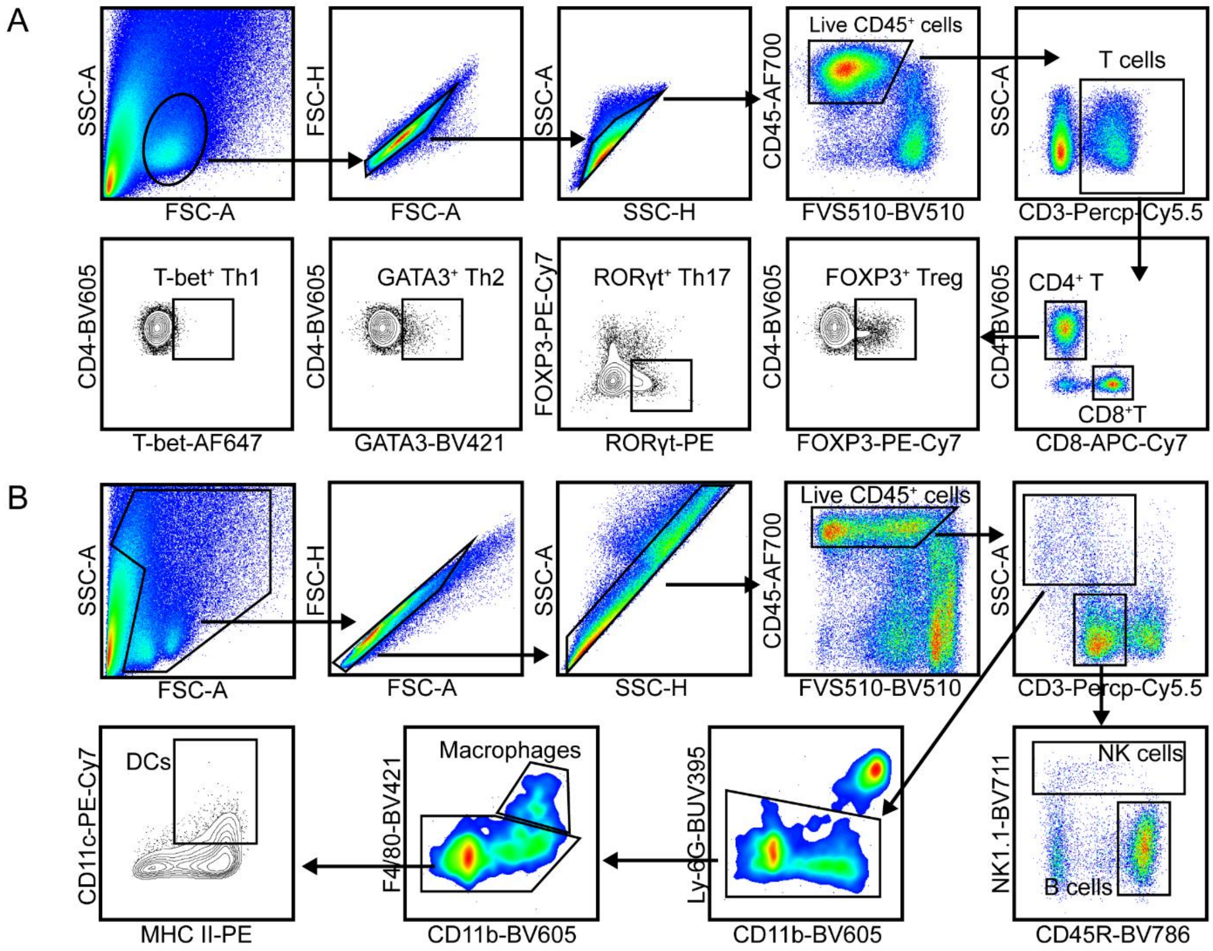

Figure S3, related to Figure 2. Gating strategy for multicolor flow cytometry

A-B, Gating strategy for multicolor flow cytometry.

Figure S4, related to Figure 2

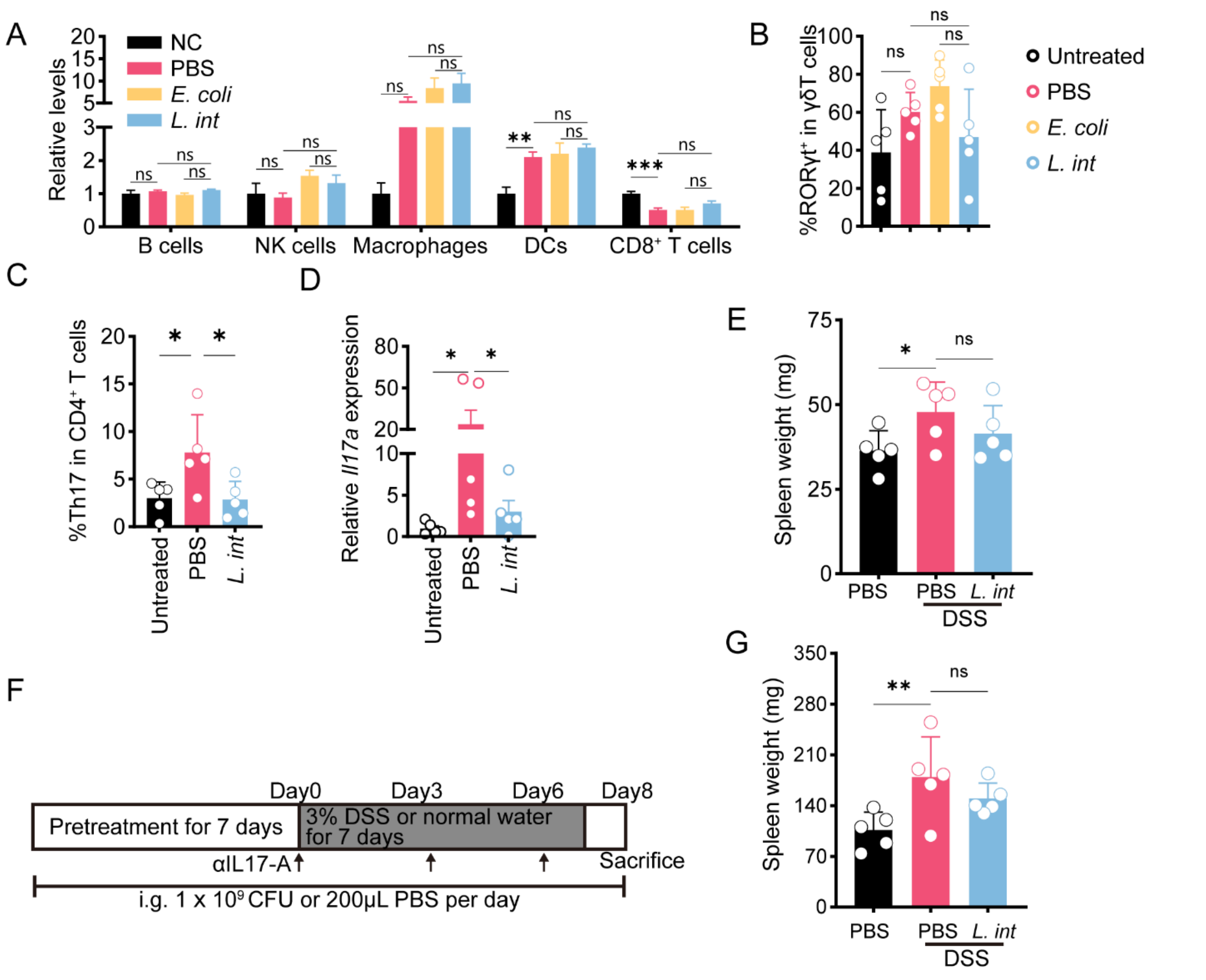

Figure S4, related to Figure 2. *L. intestinalis* relieved colitis in a Th17-dependent way.

A-B, Related levels of major immune cells **(A)** and frequencies of $TCR\gamma\delta^{+}ROR\gamma t^{+}$ T cells **(B)** were tested by multicolor flow cytometry in colon lamina propria of untreated mice and chronic DSS-treated mice with gavage of PBS, *E. coli*, or *L. intestinalis* (*L. int*). **C-D**, Frequencies of Th17 cells in colon lamina propria of untreated mice and acute DSS-treated mice with gavage of PBS or *L. intestinalis* (*L. int*) **(C)**, and *Il17a* expression were performed **(D)**. **E,** Spleen weight was compared among untreated *Rag1*$^{-/-}$ mice and acute DSS-treated *Rag1*$^{-/-}$ mice with gavage of PBS or *L. intestinalis* (*L. int*). **F,** Pretreatment with gavage of PBS or *L. intestinalis* (*L. int*) was started on the 7th day before DSS treatment and until the end of the experiment. Control group (Untreated) received PBS gavage without DSS treatment. On day 0, 3, and 6 of DSS course, all mice were intraperitoneally injected with IL-17A neutralizing antibodies (αIL17-A). **G,** Spleen weight was compared among untreated or acute DSS-treated mice with gavage of PBS or *L. intestinalis* (*L. int*), which all were administrated with αIL17-A. Error bars indicate mean ± SEM. n = 5. ns, no significance; *P < .05; **P < .01; ***P < .001. P values were based on one-way ANOVA with post-hoc test.

Figure S5, related to Figure 3

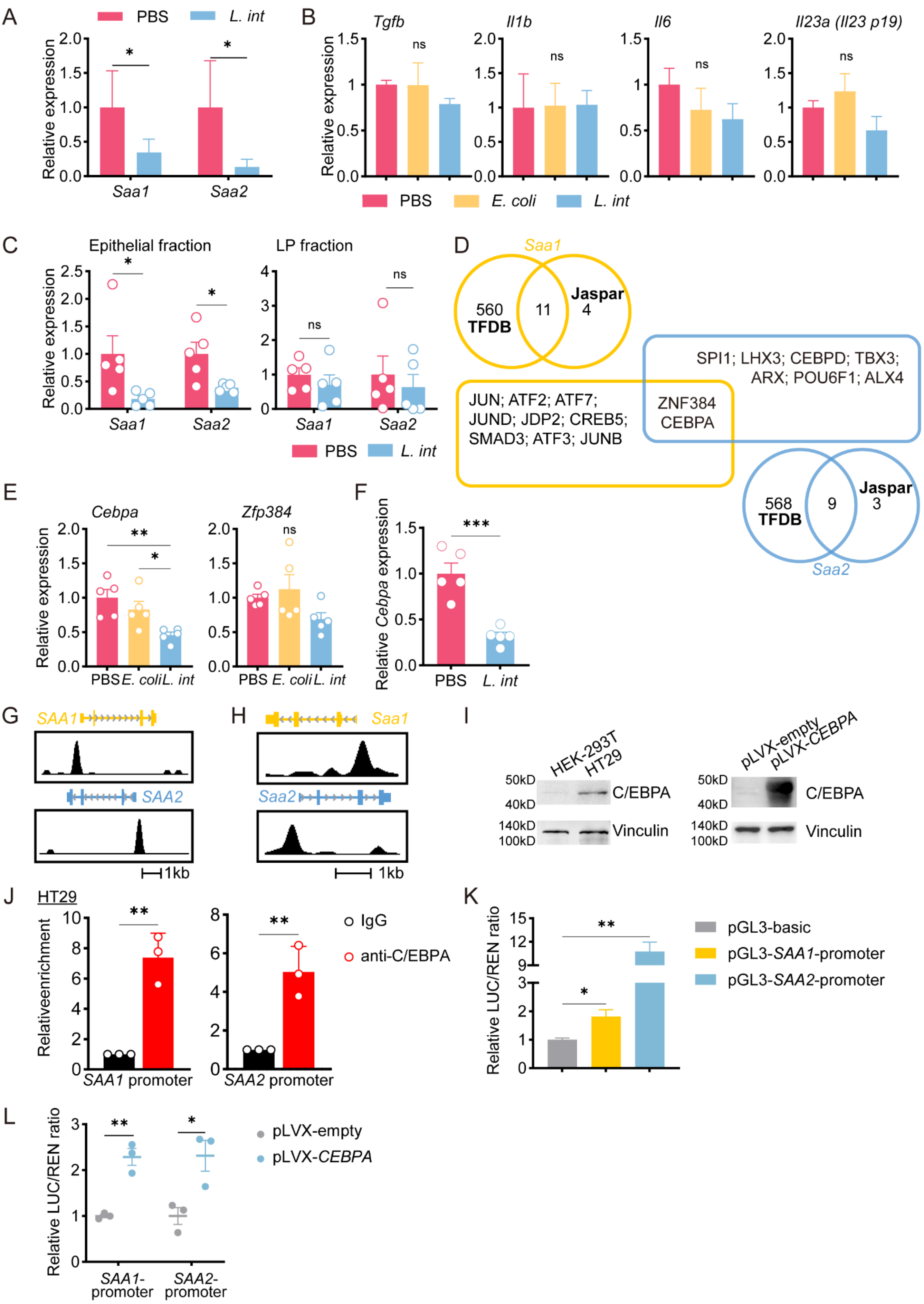

Figure S5, related to Figure 3. The colonization of *L. intestinalis* induced colonic epithelial SAA1/2 expression via CEBP/A, but did not alter cytokine profiles

A, Expression of *Saa1/2* was evaluated in bulk colon samples from acute DSS-treated mice with gavage of PBS or *L. intestinalis* (*L. int*) (n = 5). **B,** Expression of *Tgfb*, *Il1b*, *Il6* and *Il23a* was evaluated in bulk colon

samples from chronic DSS-treated mice with gavage of PBS, *E. coli* or *L. intestinalis* (*L. int*) (n = 5). **C,** Expression of *Saa1/2* was evaluated in colonic epithelial and lamina propria fractions from acute DSS-treated mice with gavage of PBS and *L. intestinalis* (*L. int*) (n = 5). **D,** The potential transcriptions of *Saa1* and *Saa2* were predicted by JASPAR and TFDB. **E,** Expression levels of *Cebpa* and *Zfp384* were evaluated in bulk colon samples from chronic DSS-treated mice with gavage of PBS, *E. coli*, and *L. intestinalis* (*L. int*) (n = 5). **F,** *Cebpa* expression was evaluated in bulk colon samples from acute DSS-treated mice with gavage of PBS and *L. intestinalis* (*L. int*) (n = 5). **G-H,** C/EBPA binding regions near the transcription start sites of human SAA1/2 **(G)** and mouse *Saa1/2* **(H)** were obtained from Cistrome Data Browser. **I,** The endogenous expression level of C/EBPA in HEK-293T and HT29 was detected by western blot (left). Overexpression of C/EBPA was tested by western blot in HEK-293T (right). **J,** The enrichment at SAA1 and SAA2 promoter was detected by CUT&RUN-qPCR in HT29 cell using anti-C/EBPA or control IgG. **K,** The luciferase activity was tested for the transcription function of *SAA1* and *SAA2* promoter-driven luciferase reporters. The firefly luciferase (LUC) was driven by SAA1 or SAA2 promoter region. The trans-activation ability of C/EBPA was measured by the LUC / Renilla luciferase (REN) ratio in HEK-293T cells co-transfected with pLVX-empty or pLVX-CEBPA (n = 3). Error bars indicate mean ± SEM. ns, no significance; **P* < .05; ***P* < .01; ****P* < .001. *P* values were based on Student's t test and one-way ANOVA with post-hoc test.

Figure S6, related to Figure 4

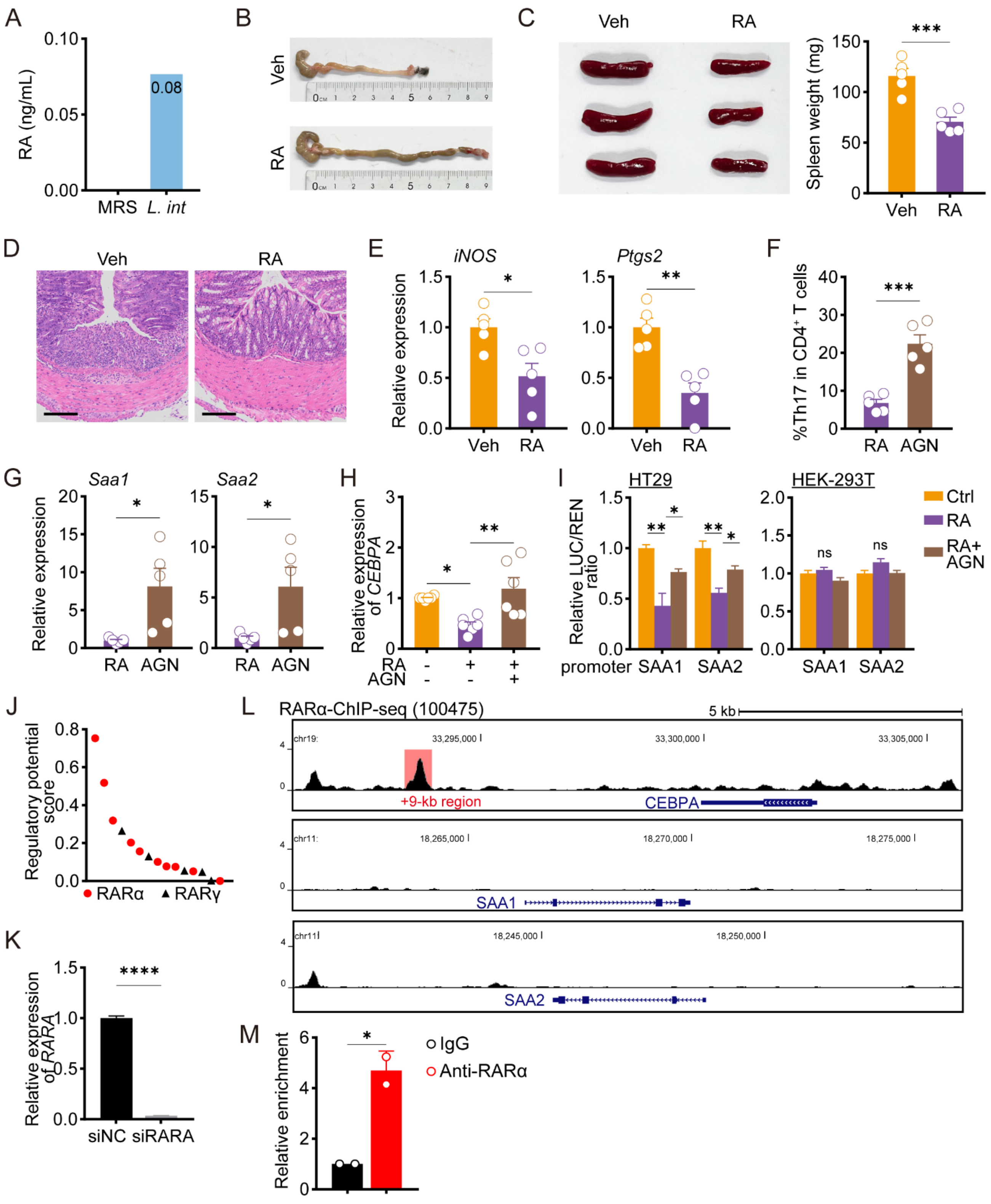

Figure S6, related to Figure 4. *L. intestinalis* contributed retinoic acid synthesis to relieve colitis.

A, Quantification of retinoic acid (RA) was performed in the MRS media or MRS media incubated with *L. intestinalis* (*L. int*). **B-E,** Representative colon images **(B)**, spleen size and weight **(C)**, pathology (scale bar, 200 μm) **(D)**, and colonic expressions of *iNOS* and *Ptgs2* **(E)** were compared between DSS-treated mice with vehicle (Veh) or retinoic acid (RA) gavage (n=5). **F-G,** Frequencies of Th17 cells in colon lamina propria **(F)** and expression of *Saa1/2* **(G)** were compared between DSS-treated mice with retinoic acid (RA) or retinoic acid + AGN193109 (AGN) administration (n=5). **H,** Expression of C/EBPA was tested in HT29 cells treated with RA or AGN (n=6). **I,** The firefly luciferase was driven by *SAA1* or *SAA2* promoters. The LUC / REN ratio was measured in HT29 and HEK-293T cells with treatment of retinoic acid (RA) or retinoic acid receptors antagonist (AGN193109, AGN). **J,** The regulatory potential score of RARs were

obtained from Cistrome Data Browser, spanning a region of ~100 kb from transcription start site of *CEBPA* gene. **K,** Expression of *RARA* was tested in HT29 cells with RARA knockdown by siRNA. **L,** RARα binding regions in the flanking regions of *CEBPA* were obtained from Cistrome Data Browser. **M,** The enrichment at +9-kb region of *CEBPA* was detected by ChIP-qPCR in HT29 cells using anti-RARα or control IgG. Error bars indicate mean ± SEM. ns, no significance; **P* < .05; ***P* < .01; ****P* < .001. *P* values were based on Student's t test and one-way ANOVA with post-hoc test.

Figure S7, related to Figure 5

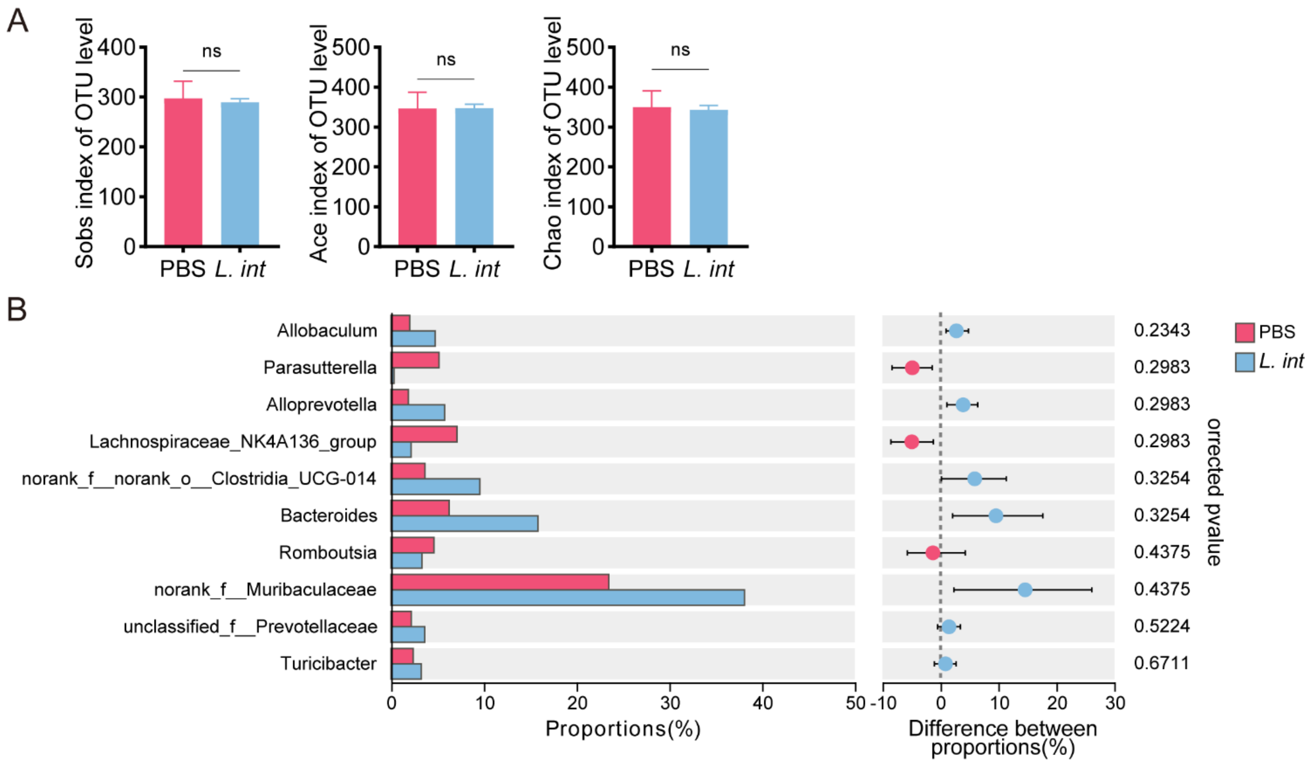

Figure S7, related to Figure 5. *L. intestinalis* promoted retinoic acid synthesis through its own ALDH and by enhancing host ALDH

A, The α-diversity of the fecal was compared between chronic DSS-treated mice with PBS gavage and with *L. intestinalis* (*L. int*) gavage (n = 5). **B,** The genus-level taxonomic compositions with top 10 P value were compared between chronic DSS-treated mice with PBS gavage and with *L. intestinalis* (*L. int*) gavage using Wilcoxon rank-sum test with corrected P value. *P* values were based on Student's t test and Wilcoxon rank-sum test with corrected P value.

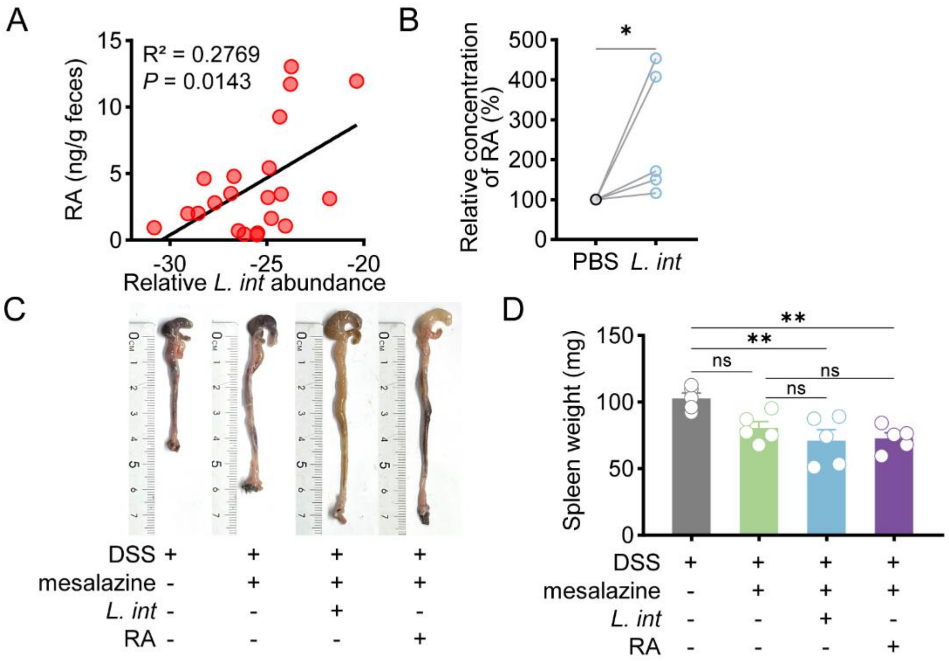

Figure S8, related to Figure 6. *L. intestinalis* suppressed the CEBP/A-SAA1/2-Th17 axis in UC patients and exerted therapeutic effect on DSS-induced colitis.

A, The correlation analysis was performed in feces from IBD patients to determine the relationship between the retinoic acid (RA) concentration and *L. intestinalis* (*L. int*) abundance. **B**, The relative concentration of RA was measured in supernatant of fecal *in vitro* anaerobic fermentation administrated with or without *L. intestinalis* (*L. int*). **C-D,** Representative colon images **(C)** and spleen size and weight **(D)** were compared DSS-treated mice treated with vehicle (Veh), or mesalazine alone or in combination with *L. intestinalis* (*L. int*) or retinoic acid (RA) (n=5). Error bars indicate mean ± SEM. ns no significant; **P* < .05; ***P* < .01. *P* values were based on Pearson correlation test, ratio paired t test, and one-way ANOVA with post-hoc test.
